# Editorial: Insights in computational genomics: 2022

**DOI:** 10.3389/fgene.2023.1256011

**Published:** 2023-07-24

**Authors:** Richard D. Emes, Mehdi Pirooznia, Quan Zou, Marco Pellegrini

**Affiliations:** ^1^ Nottingham Trent University, Nottingham, United Kingdom; ^2^ School of Medicine, Johns Hopkins University, Baltimore, MD, United States; ^3^ Pharmaceutical Data Sciences, R&D Johnson & Johnson, Boston, MA, United States; ^4^ Institute of Fundamental and Frontier Sciences, University of Electronic Science and Technology of China, Chengdu, China; ^5^ Consiglio Nazionale delle Ricerche, Pisa, Italy

**Keywords:** computational genomics, cancer studies, single cell RNA-seq technology, human genomic variation, deep learning

The goal of this Research Topic is to shed light on the progress made in the past decade in the Computational Genomics field, to gauge its future challenges, and to provide a thorough overview of the field’s current status. We hope that this article Research Topic will inform, inspire and provide guidance to researchers in the field. Foundational Research Topic ranging from still unsolved evolutionary mechanisms at the genomic level and the challenges posed by human genomic variation are powerful drivers of current and future research in Computational Genomics. The challenge that genomics poses to computational theory is to be highlighted, ranging from the role of Artificial Intelligence and Deep Learning (DL) in this context to the likely impact of emerging Single Cell RNA Sequencing (scRNA-Seq) methodologies in transcriptomics. Steady progress in tools for automated managing of large heterogeneous genomic and biological data is likely to bring good dividends in the near future. Specific applications of computational genomics support cancer studies for tasks such as drug repositioning and finding the role of immune system genes in cancer. Also, computational genomics is a key helper to plant science in the effort to cope with the effects of climate changes in the long run, with global food security as a goal.

Here is an overview of the issues presented in this Research Topic.

The evolution of genomes and codon encodings is a source of key fundamental questions still needing an answer. In this area, Belinky et al. highlight major differences between prokaryotes and eukaryotes regarding the double substitutions of nucleotides in codon encodings.

Dong et al. provide experimental evidence on the performance of recently developed DL methods compared to more traditional flavors of Machine Learning (ML) when applied to the prediction of risk in cancer. Using a very large cohort of patients and three cancer test types, they give interesting hints for further research on this Research Topic.

The Human Genome Project (HGP) lasted from 1990 to 2003 and has brought about a scientific revolution in genomics of the type the philosopher Thomas S. Kuhn has described. As with every scientific revolution, its long-lasting value is that of posing new questions. Singh et al. argue that the Human Pangenome Project is the next logical step on the road opened by the HGP, allowing us to reach new heights in genomic research in the near future.

Single Cell RNA Sequencing is one of the key new technologies in transcriptomics, enabling a finer view of the diverse roles of single cells (or cell types) within a sample. New technologies often need new algorithmic insights and robust statistical filtering methodologies. Li et al. propose in their article a novel method for detecting a limited number of representative cells within a pool of thousands (up to hundreds of thousands) of cells typically analyzed in a single scRNA-Seq run. Carangelo et al. give an overview of the scRNA-Seq technologies with particular emphasis on the scalability of the algorithmic analytic tools needed to cope with the ever-increasing rate of data generation. Interestingly the field of scRNA-Seq research is poised to move from a niche sector to a broader role in a clinical setting for human health.

Modern metabolomics assays produce vast volumes of complex data. Thus, there is a growing opportunity for the application of Machine Learning to analyze such data, recognize new patterns, and build models across multiple levels (from the genomic to the metabolic aspects). Galal et al. give an overview of this burgeoning area of research with emphasis on its potential for leading to new disease classifications and to uncovering key aspects of the disease onset and progression.

Gene duplication and gene transfer are important evolutionary mechanisms still needing further research and attention. Here Zhang et al. give an overview of the current trends and resources in the fascinating subject of intra-species detection of gene duplications, which is often a key strategy in solving the intra-species functional gene annotation problem. Nayar et al. study the phenomenon of horizontal gene transfer mediated by conjugation, which is considered an important evolutionary mechanism of bacteria. With the new proposed methodology (ggMOB) they found that over half of the bacterial genomes contained one or more known conjugation features that matched exactly to at least one other genome. Science and technology often require a constant and valuable background activity of standardization and unification of procedures and data. Maia et al. describe a computational workflow (AnnotaPipeline) for the annotation of eukaryotic proteins using multi-omics data aiming at overcoming problems due to the variety of sequencing platforms that generate increasing amounts of data, thus making manual annotation no longer feasible.

Cancer studies have been and still are at the forefront of the application of genomics to human health. In this context, we report two studies: Ai et al. identify genes involved in the immune response to colorectal cancer for the purpose of cancer prognosis and potential impact on future immune therapies, while Bennett et al. compare the gene expression profiles of disease-states with the perturbation on gene-expression profiles by a given drug and are thus able to identify 24 existing drugs with potential beneficial effects for patients of Esophageal Cancer (EC).

As climate and climate-related phenomena are gaining continuous attention in public discourse and long-term planning, promoting plant genomics studies is strategic for the future of food security. Here we report two exemplary applicative studies in plant genomics. Jiang et al. analyze the transcriptional dynamics of filling stage Tartary buckwheat seeds in order to provide a theoretical basis for improving the yield of Tartary buckwheat. Pan et al. investigate wheat genotypes and differential gene expression in several climate-related conditions to highlight and clarify the cold-resistance mechanisms leading to potentially higher yields in uncertain climatic conditions.

